# Monogeneans in intergeneric hybrids of leuciscid fish: Is parasite infection driven by hybrid heterosis, genetic incompatibilities, or host-parasite coevolutionary interactions?

**DOI:** 10.1186/s12983-022-00481-w

**Published:** 2023-01-26

**Authors:** Neira Dedić, Lukáš Vetešník, Andrea Šimková

**Affiliations:** 1 Department of Botany and Zoology, Faculty of Science, Masaryk University, Kotlářská 2, 611 37 Brno, Czech Republic; 2 Institute of Vertebrate Biology, Czech Academy of Sciences, Květná 8, 603 65 Brno, Czech Republic

**Keywords:** Monogeneans, Host specificity, Freshwater fish, Hybrids, Hybrid heterosis, Genetic incompatibilities, Host-parasite coevolution

## Abstract

**Background:**

Several hypotheses have been proposed to explain parasite infection in parental species and their hybrids. Hybrid heterosis is generally applied to explain the advantage for F1 generations of hybrids exhibiting a lower level of parasite infection when compared to parental species. Post-F1 generations often suffer from genetic incompatibilities potentially reflected in the higher level of parasite infection when compared to parental species. However, the presence of specific parasites in an associated host is also limited by close coevolutionary genetic host-parasite associations. This study focused on monogenean parasites closely associated with two leuciscid fish species—common bream and roach—with the aim of comparing the level of monogenean infection between parental species and hybrids representing two F1 generations with different mtDNA and two backcross generations with different cyto-nuclear compositions.

**Results:**

Monogenean infection in F1 generations of hybrids was lower when compared to parental species, in line with the hybrid heterosis hypothesis. Monogenean infection in backcross generations exhibited similarities with the parental species whose genes contributed more to the backcross genotype. The distribution of monogeneans associated with one or the other parental species showed the same asymmetry with a higher proportion of roach-associated monogeneans in both F1 generations and backcross generation with roach in the paternal position. A higher proportion of common bream-associated monogeneans was found in backcross generation with common bream in the paternal position.

**Conclusions:**

Our study indicated that cyto-nuclear incompatibilities in hybrids do not induce higher monogenean infection in backcross generations when compared to parental species. However, as backcross hybrids with a higher proportion of the genes of one parental taxon also exhibited high level of this parental taxon-associated parasites, host-parasite coevolutionary interactions seem to play an obvious role in determining the level of infection of host-specific monogeneans in hybrids.

## Introduction

Interspecies hybridization is limited by the varieties of prezygotic and postzygotic isolation mechanisms [1]. However, this phenomenon naturally appears in different animal taxa (e.g. [2–15]); among vertebrate species, hybrids are mostly reported in fish. Cyprinoid fish (formerly considered as cyprinids, see Schönhuth et al. [16]) exhibit the highest hybridization rate [2].

Positive heterosis or hybrid vigor is commonly documented in F1 generations of hybrids, which exhibit superior vigour-related traits in comparison to both parents [3–5]. However, hybridization breaks up the functional networks of genes with unique allelic combinations (i.e., coadapted gene complexes) by bringing together combinations of alleles at different loci that have not been “tested” together by evolution [4, 6]. Such disruption of coadapted gene complexes or so-called genetic incompatibility, as defined by Dobzhansky [17] and Muller [18], often results in hybrid breakdown expressed by deleterious phenotypes associated with reduced viability and fecundity (often sterility), developmental abnormalities, and low immunity performance (e.g., [5–8]).

Hybrid breakdown, regarded as an apparent reduction in fitness, has been documented in post-F1 generations (backcross and F2 generations have been the most commonly investigated) (e.g., [5, 6, 8, 9]). A tidal pool-inhabiting copepod, *Tigriopus californicus*, has been extensively studied with respect to hybridization (e.g., [3, 4, 10–12]). Interpopulation hybrids of F1 generations of this copepod were shown to exhibit heterosis, whilst post-F1 generations, despite being viable and fertile, suffered from declining fitness [3, 12, 13]. Concerning fish, Stelkens et al. [8] showed that F2 hybrids of haplochromine cichlids consistently exhibited reduced viability (survival) and a loss of fitness of up to 43% when compared to non-hybrid crosses and up to 21% when compared to F1 hybrids. In incipient species of normal and dwarf whitefish (*Coregonus clupeaformis*), Renaut et al. [14] showed high gene misexpression in backcross hybrids when compared to F1 hybrids at the embryonic and juvenile stages.

Interactions within cells that lead to molecular incompatibilities in hybrids take place either at the structural (protein–protein interactions) or regulatory (gene–gene interactions) level [19, 20]. Such incompatibilities can arise due to different inheritance patterns between the organelle genome (mostly uniparental) and the nuclear genome (biparental) [6, 21, 22]. Therefore, paternal (inter-mitotype) backcrosses have mismatched mitochondrial and nuclear genomes in contrast to maternal (intra-mitotype) backcrosses [6]. Ellison and Burton [5] showed that the disruption of nuclear-mitochondrial gene interactions can account for the reduced fitness of interpopulation hybrids of *T. californicus*; specifically, they showed that maternal backcross hybrids exhibited the recovery of mitochondrial ETS function and the concomitant recovery of fitness and survival. In fish, cytonuclear incompatibility was suggested as one of the potential explanations of asymmetrical hybrid viability in reciprocal crosses [23], and a factor affecting reproductive isolation (more specifically, directional assortative mating through behavioral traits) [24, 25].

Parasite load is an important measure of host vigour reflecting heterosis or hybrid breakdown in fish hybrids [26]. Studies focused on metazoan parasite infection in wild living cyprinoid fish and their intergeneric hybrids showed that F1 hybrids harbor metazoan parasites of both pure (parental) species, especially gill and fin monogeneans specific to one or the other parental species [7, 27, 28]. The level of parasite infection was lower in F1 hybrids than in each of the two parental fish species, which is in line with the general hypothesis of heterosis advantage in the F1 generation of hybrids [29] and the hybrid resistance scenario of parasite infection [30]. Fritz et al. [30] proposed four static scenarios to explain the pattern of resistance and susceptibility to parasites in hybrids and parental species: (1) the additive scenario, predicting that resistance to parasites in hybrids is similar to the average resistance of the parental taxa, (2) the dominance scenario, predicting that resistance to parasites in hybrids is similar to that of one of the parental taxa, (3) the hybrid resistance scenario, predicting that hybrids have a higher resistance to parasites when compared to both parental taxa, and (4) the hybrid susceptibility scenario, predicting higher susceptibility to parasites for hybrids than for parental taxa. Concerning parasites in fish hybridizing system, Dupont and Crivelli [31] showed high metazoan parasite load in the hybrids of cyprinoid species under the conditions of a high frequency of hybrids, which corresponds to the hybrid susceptibility scenario proposed by Fritz et al. [30] and also supports the dynamic scenario of parasite infection in pure species and their hybrids introduced by Wolinska et al. [32]. The dynamic scenario is based on the prediction that the most common host genotype is a target of parasite adaptation, and that negative frequency-dependent selection is a mechanism generating changes in parasite load in the genotypes of pure species and their hybrids over time.

Host-specific parasite species (especially ectoparasitic gill and skin monogeneans in fish hosts) are associated with one host fish species or with a restricted range of phylogenetically closely-related (often congeneric) fish species [33, 34]. From the coevolutionary host-parasite perspective, reciprocal genetic coadaptation is hypothesized between the host genotype and the associated parasite [35, 36]; the strength of host-parasite coadaptation should be more evidenced for host-specific parasites (i.e. parasite species associated with a single host species as a result of long-term coadaptation). The hybridization of evolutionarily distant parental genomes (such is a case of the hybridization of two cyprinoid species, common bream (*Abramis brama*) and roach (*Rutilus rutilus*) [27]) is rare. If this hybridization occurs, then it results in gene breakdown in defense mechanisms, especially when the interaction between host and parasite exhibits high specificity [37–39]. The high susceptibility of hybrids to specific parasites was observed in hybrid zones of mice and was explained by breakdown in the host-parasite genetic coadaptation system due to host hybridization [40, 41]. However, Baird et al. [15], using an extensive data set, showed that hybrid mice exhibit reduced parasite diversity and load, and indicated that host immune genes tracking parasites (predicted by the Red Queen hypothesis) escape Dobzhansky-Muller genetic incompatibilities, generating hybrid variants untargeted by parasites.

Common bream (*Abramis brama*) and roach (*Rutilus rutilus*) are two non-congeneric evolutionarily divergent leuciscid species (belonging to cyprinoids). These fish species exhibit different phenotypes and ecology; however, they have the same spawning period and similar ecological requirements [42]. Each of common bream and roach harbor unique metazoan parasite species, especially concerning their specific monogeneans parasitizing gills [43]. F1 hybrids harbored all parasite species infecting common bream or roach; however, the level of parasite infection was low in hybrids when compared to pure species, this fact supporting the hybrid heterosis advantage hypothesis [27]. This finding is also in line with the hybrid resistance scenario (one of the four static scenarios of parasite infection in parental species and hybrids proposed by Fritz et al. [30]). In addition, F1 hybrids of common bream and roach expressed intermediate immune gene diversity, specifically major histocompatibility complex (MHC) diversity, which may be considered as the optimal MHC diversity potentially associated with low total parasite load [44]. However, our previous finding opened up new questions about the levels of infection by host-specific parasites in post-F1 generations of common bream and roach, as such generations may suffer from genetic incompatibilities, and, at the same time, different post F1-generations exhibit different proportions of parental genes – specifically, those parental (i.e., pure species) genotypes likely evolved in coadaptation with host-specific parasites. F1 hybrids have been widely found in the natural habitats of common bream and roach (e.g., [27, 45–47]). However, in some studies, backcross and F2 hybrids were shown to occur in only low frequencies [46, 48, 49], suggesting the low fitness of F1 hybrids when compared to pure species. Some studies even revealed the complete absence of specimens representing post-F1 generations in natural habitats [27, 47, 50].

In the light of previous evidence, the aim of our study was to analyze the presence and infection levels of monogenean species in common bream and roach and in their hybrids, these representing two F1 generations (with different mtDNA) and two backcross generations with different levels of cyto-nuclear interactions, all fish were obtained by artificial breeding. F1 generations were obtained by crosses of wild living common bream and roach. Backcrosses were performed between females of natural F1 hybrids with common bream mtDNA and males of common bream (backcrosses within common bream mtDNA), or males of roach (backcrosses between roach and common bream mtDNA). We hypothesized hybrid heterosis for the F1 generations, as previously documented by Krasnovyd et al. [27] (i.e. lower parasite infection in F1 hybrids when compared to each parental species) and hybrid breakdown for the backcross generations (i.e. higher parasite infection in backcross hybrids when compared to parental species). Next, we compared two backcrosses generations hypothesized differences in monogenean infection between intra-mitotype backcrosses (parents with the same, i.e. common bream mtDNA) and inter-mitotype backcrosses (parents with different, i.e. common bream and roach mtDNA) due to the different level of cyto-nuclear incompatibilities. Finally, we tested whether distribution of parental species-specific parasites is affected by mtDNA in F1 hybrids, and by different cyto-nuclear composition in backcross hybrids.

## Results

### Parasite species richness in parental and hybrid lines

Roach harbored 12 monogenean species including 9 *Dactylogyrus* species, 2 *Gyrodactylus* species, and *Paradiplozoon homoion*. Common bream harbored 8 monogenean species including 4 *Dactylogyrus* species, 3 *Gyrodactylus* species, and *Paradiplozoon homoion* (Table 1). Only 3 monogenean species (*G. vimbi*, *G. carassi* and *P. homoion*) were shared between roach and common bream. Both F1 generations of hybrids and the backcross generation with roach in paternal position harbored 15 monogenean species, whilst the backcross generation with common bream in paternal position harbored 14 monogenean species (Tables 1, 2). The majority of representatives within each of the F1 generations harbored both monogenean species associated with roach and monogenean species associated with common bream (specifically, monogeneans of both parental species were present in 77% of specimens representing F1 hybrids with roach mtDNA, and 73% of specimens representing F1 hybrids with common bream mtDNA). In the case of the backcross generation with paternal roach, only 46% of specimens harbored monogenean species of both species, the others lacking monogenean species of common bream. In the case of the backcross generation with paternal common bream, 77% of specimens harbored monogeneans of both species, the others lacking monogenean species of roach. Using the Jaccard index, the monogenean communities of roach and common bream were highly divergent (0.176), whilst the overall similarities in monogenean communities between pairs of hybrid generations were high (0.813). Concerning the similarities in monogenean communities between parental generations and hybrid generations, the highest similarities were found between roach and F1 generation with roach mtDNA or the backcross generation with paternal roach (0.688), and between common bream and the backcross generation with paternal common bream (0.467).

**Table 1:** Monogenean abundance (A, mean ± SD), prevalence (P) and intensity of infection (II, min–max) for each of fish groups (*Rutilus rutilus*, *Abramis brama*, F1 generation with roach mtDNA (F1 *R. rutilus* × *A. brama*), F1 generation with common bream mtDNA (F1 *A. brama* × *R. rutilus*))

Parasite species	*R. rutilus*			*A. brama*			F1 *R. rutilus* x *A. brama*			F1 *A. brama* x *R. rutilus*		
	A	P (%)	II (min–max)	A	P (%)	II (min–max)	A	P (%)	II (min–max)	A	P (%)	II (min–max)
*D. crucifer*	108.44 ± 115.38	100	6–374	–	–	–	13.77 ± 13.13a*	77	6–37a*	23.13 ± 27.09a*	80	2–94
*D. caballeroi*	5.44 ± 12.29	44	1–48	–	–	–	2.00 ± 5.79	23	2–21	0.07 ± 0.26	7b	1
*D. rutili*	5.94 ± 9.93	72	1–33	–	–	–	2.38 ± 3.80	54	1–11	8.53 ± 15.78	67	1–57
*D. nanus*	8.00 ± 9.78	83	1–33	–	–	–	1.38 ± 1.71a*	46b	1–4	5.60 ± 9.86	60	2–33
*D. suecicus*	4.28 ± 11.79	39	1–48	–	–	–	0.62 ± 1.04	31	1–3	0.40 ± 1.06	20	1–4
*D. fallax*	1.22 ± 2.56	33	1–10	–	–	–	0.31 ± 0.48	31	1a	0.27 ± 1.03	7	4
*D. similis*	3.78 ± 5.34	56	1–16	–	–	–	0.31 ± 0.63	23	1–2	–	–	–
*D. sphyrna*	3.83 ± 5.27	61	1–18	–	–	–	0.23 ± 0.59a	15b	1–2	0.60 ± 1.45a	20b	1–5
*D. rarissimus*	2.17 ± 4.89	28	4–20	–	–	–	–	–	–	0.20 ± 0.77	7	3
*D. auriculatus*	–	–	–	21.08 ± 45.40	100	2–171	–	–	–	0.47 ± 1.36d*	13e*	2–5
*D. wunderi*	–	–	–	13.69 ± 14.01	92	1–41	0.46 ± 0.97c*	23d*	1–3	0.87 ± 1.88d*	27e*	2–7
*D. zandti*	–	–	–	174.15 ± 145.69	100	30–471	3.62 ± 3.59c*	69d	1–11c*	1.80 ± 3.32d*	53e*	1–13e*
*D. falcatus*	–	–	–	15.00 ± 12.35	85	4–37	0.38 ± 1.39c*	8d*	5	1.40 ± 2.41d*	40e	1–7e*
*G. vimbi*	47.06 ± 101.19	78	1–398	8.23 ± 11.25	62	2–36	4.85 ± 7.22a	46	2–21a	7.67 ± 11.16	67	1–40
*G. carassii*	7.94 ± 14.72	61	1–52	0.23 ± 0.83	8	3	0.31 ± 0.63a	23b	1–2	–	–	–
*G. elegans*	–	–	–	1.92 ± 3.20	38	1–10	0.38 ± 0.77	23	1–2	5.13 ± 11.84	47	1–46
*P. homoion*	12.72 ± 19.74	50	1–67	2.00 ± 4.26	31	1–12	1.00 ± 1.78	31	1–5a	2.67 ± 8.50	20	2–33

^a^Significant differences between *R. rutilus* and F1 generation revealed by Mann–Whitney test, ^b^significant differences between *R. rutilus* and F1 generation revealed by Fisher exact test, ^c^significant differences between *A. brama* and F1 generation revealed by Mann–Whitney test, ^d^significant differences between *A. brama* and F1 generation revealed by Fisher exact test, asterisks indicate significant difference after Bonferroni correction

**Table 2:** Parasite abundance (A, mean ± SD), prevalence (P) and intensity of infection (II, max–min) for each group of fish groups ((*Rutilus rutilus*, *Abramis brama*, backcross generation with roach in paternal position (abbreviated as backcross *R. rutilus*), backcross generation with common bream in paternal position (abbreviated as backcross *A. brama*))

	*R. rutilus*			*A. brama*			Backcross *R. rutilus*			Backcross *A. brama*		
Parasite species	A	P (%)	II (min–max)	A	P (%)	II (min–max)	A	P (%)	II (min–max)	A	P (%)	II (min–max)
*D. crucifer*	108.44 ± 115.38	100	6–374	–	–	–	87.93 ± 56.02	100	3–188	7.15 ± 12.72a*	54b*	1–36a*
*D. caballeroi*	5.44 ± 12.29	44	1–48	–	–	–	4.40 ± 3.74	67	3–10	0.77 ± 1.36	31	1–4
*D. rutili*	5.94 ± 9.93	72	1–33	–	–	–	4.27 ± 6.05	67	1–19	1.08 ± 1.89a	31b	2–3
*D. nanus*	8.00 ± 9.78	83	1–33	–	–	–	2.53 ± 4.60a	40b	1–12	0.46 ± 1.20a*	15b*	2–4
*D. suecicus*	4.28 ± 11.79	39	1–48	–	–	–	1.13 ± 1.51	47	1–5	0.15 ± 0.55	8	2
*D. fallax*	1.22 ± 2.56	33	1–10	–	–	–	–	–	–	–	–	–
*D. similis*	3.78 ± 5.34	56	1–16	–	–	–	1.13 ± 1.51	40	2–4	0.23 ± 0.60a	15b	1–2
*D. sphyrna*	3.83 ± 5.27	61	1–18	–	–	–	3.33 ± 4.70	47	2–14	0.54 ± 1.05a	23b	2–3
*D. rarissimus*	2.17 ± 4.89	28	4–20	–	–	–	0.73 ± 1.33	27	2–4a	–	–	–
*D. auriculatus*	–	–	–	21.08 ± 45.40	100	2–171	0.13 ± 0.52c*	7d*	2f*	17.69 ± 19.17	77	2–57
*D. wunderi*	–	–	–	13.69 ± 14.01	92	1–41	–	–	–	14.85 ± 22.65	85	1–83
*D. zandti*	–	–	–	174.15 ± 145.69	100	30–471	0.47 ± 0.92c*	27d*	1–3c*	42.38 ± 39.70c*	100	5–146c*
*D. falcatus*	–	–	–	15.00 ± 12.35	85	4–37	0.13 ± 0.35c*	13d*	1c	12.92 ± 13.77	85	2–40
*G. vimbi*	47.06 ± 101.19	78	1–398	8.23 ± 11.25	62	2–36	63.00 ± 124.94	67	6–496c	31.92 ± 65.61	85	1–238
*G. carassii*	7.94 ± 14.72	61	1–52	0.23 ± 0.83	8	3	0.13 ± 0.52a	7b*	2	–	–	–
*G. elegans*	–	–	–	1.92 ± 3.20	38	1–10	1.47 ± 5.68	7d	22	5.08 ± 17.41	23	1–63
*P. homoion*	12.72 ± 19.74	50	1–67	2.00 ± 4.26	31	1–12	1.27 ± 2.63	27	2–8a	0.85 ± 1.72	23	2–5

^a^Significant differences between *R. rutilus* and backcross generation revealed by Mann–Whitney test, ^b^significant differences between *R. rutilus* and backcross generation revealed by Fisher exact test, ^c^significant differences between *A. brama* and backcross generation revealed by Mann–Whitney test, ^d^significant differences between *A. brama* and backcross generation revealed by Fisher exact test, asterisks indicate significant difference after Bonferroni correction

### Parasite load in parental and hybrid lines

*Dactylogyrus crucifer* was the species with the maximum prevalence and the highest abundance and intensity infection on roach, followed by *D. nanus* and *D. rutili* (based on abundance and prevalence). *Gyrodactylus vimbi* also reached very high abundance and intensity of infection on roach. *Dactylogyrus zandti* and *D. auriculatus* were the species with maximum prevalence on common bream. *Dactylogyrus zandti* reached the highest abundance and intensity of infection on common bream. Concerning the hybrids of each F1 generation, *D. crucifer* was also the species with the highest prevalence, abundance and intensity of infection, followed by *D. rutili*. *D. nanus*, *D. zandti* and *G. vimbi*. Concerning backcross generations, the parasite species achieving maximum prevalence and highest abundance were *D. crucifer* in the backcross generation with roach in paternal position*,* and *D. zandti* in the backcross generation with common bream in paternal position. *Dactylogyrus* spp. specific to roach had higher prevalence, abundance and intensity of infection on the backcross generation with roach in paternal position, whilst *Dactylogyrus* spp. specific to common bream had higher prevalence, abundance and intensity infection on the backcross generation with common bream in paternal position. *Gyrodactylus vimbi* was the most abundant among *Gyrodactylus* species found on both backcross generations; however, its abundance and maximum intensity of infection were higher in the backcross generation with roach in paternal position. Significant differences in prevalence, abundance and intensity of infection between each of the F1 generations of hybrids and roach or common bream are shown in Table 1, and between each of the backcross generations and roach or common bream are shown in Table 2. The parasite prevalence, abundance and maximum intensity of infection of many monogenean species was higher in parental species when compared to hybrids of F1 generations or backcross generations (see Tables 1, 2 for the significance of the MW test for abundance and intensity of infection, and the Fisher exact test for prevalence). However, the levels of infection of individual monogenean species exhibited only a few difference between roach and the backcross generation with roach in paternal position, and between common bream and the backcross generation with common bream in paternal positon.

### Heterosis versus breakdown in hybrids

**Fig. 1:**
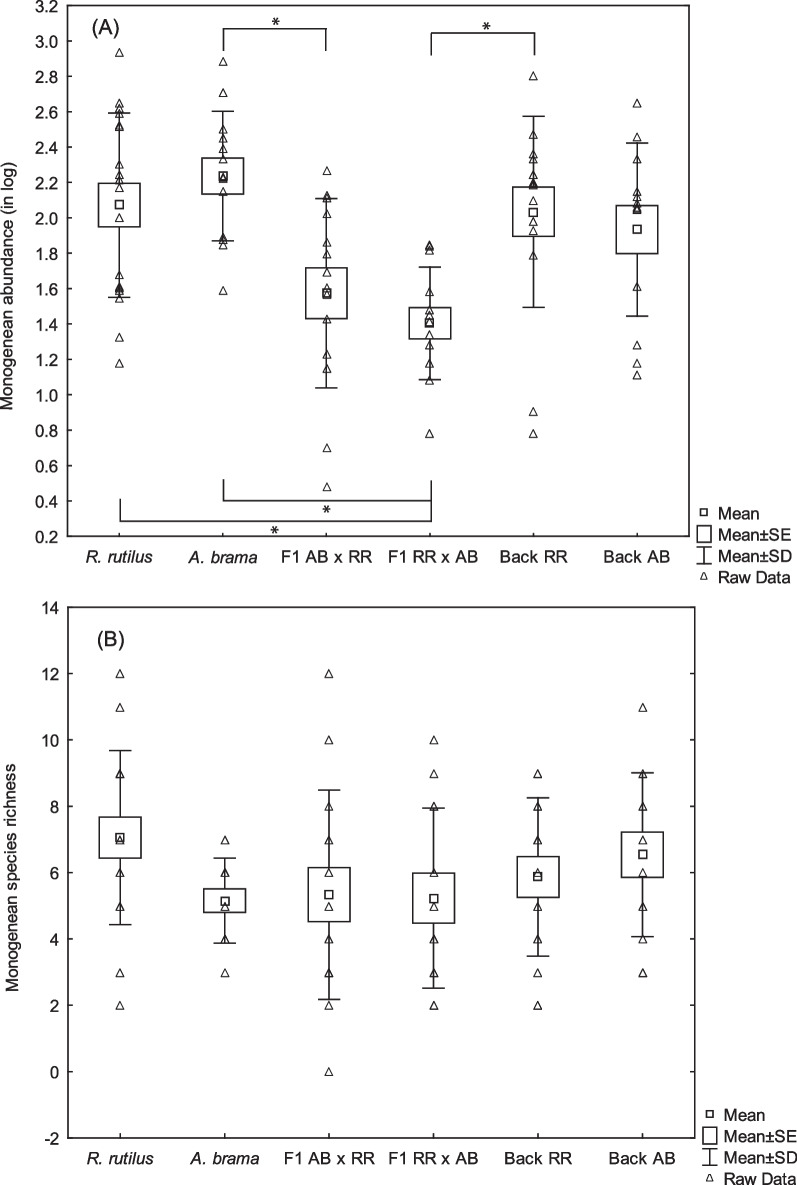
Monogeneans in pure species, F1 generations and backcross generations of hybrids. Monogenean abundance (**A**) and species richness (**B**) on *A. brama*, *R. rutilus*, F1 generation of hybrids with *A. brama* mtDNA (F1 AB × RR), F1 generation of hybrids with *R. rutilus* mtDNA (F1 RR × AB), backcross generation with *A. brama* mtDNA and paternal *R. rutilus* (Back RR), backcross generation with *A. brama* mtDNA and paternal *A. brama* (Back AB); AB—*A. brama*, RR—*R. rutilus*. Significant difference between fish groups are shown by asterisks

Significant effect of fish group on monogenean abundance was found (KW test, H(5, N = 87) = 26.851, *p* < 0.001). F1 hybrids with roach mtDNA exhibited lower monogenean abundance when compared to roach (*p* = 0.006) or common bream (*p* < 0.001). F1 hybrids with common bream mtDNA exhibited lower monogenean abundance when compared to common bream (*p* = 0.011), the monogenean abundance in F1 hybrids with common bream mtDNA tended to be higher when compared to roach; however, the difference was not statistically significant (0.089) (Fig. 1A). F1 hybrids tented to be less parasitized when compared to backcrosses; however, only the difference between F1 hybrids with roach mtDNA and backcross hybrids with paternal *R. rutilus* was significant (*p* = 0.011).

No significant effect of fish group on monogenean species richness was found (KW test, *p* = 0.207), even if roach tended to exhibit higher species richness at the level of individual fish than F1 hybrids and common bream (Fig. 1B). No significant differences in monogenean species richness between the pairs of fish groups including roach, common bream, F1 hybrids and the backcross hybrids were found (*p* > 0.05) (Fig. 1B).

### Asymmetry in host-specific parasites in hybrids

**Fig. 2:**
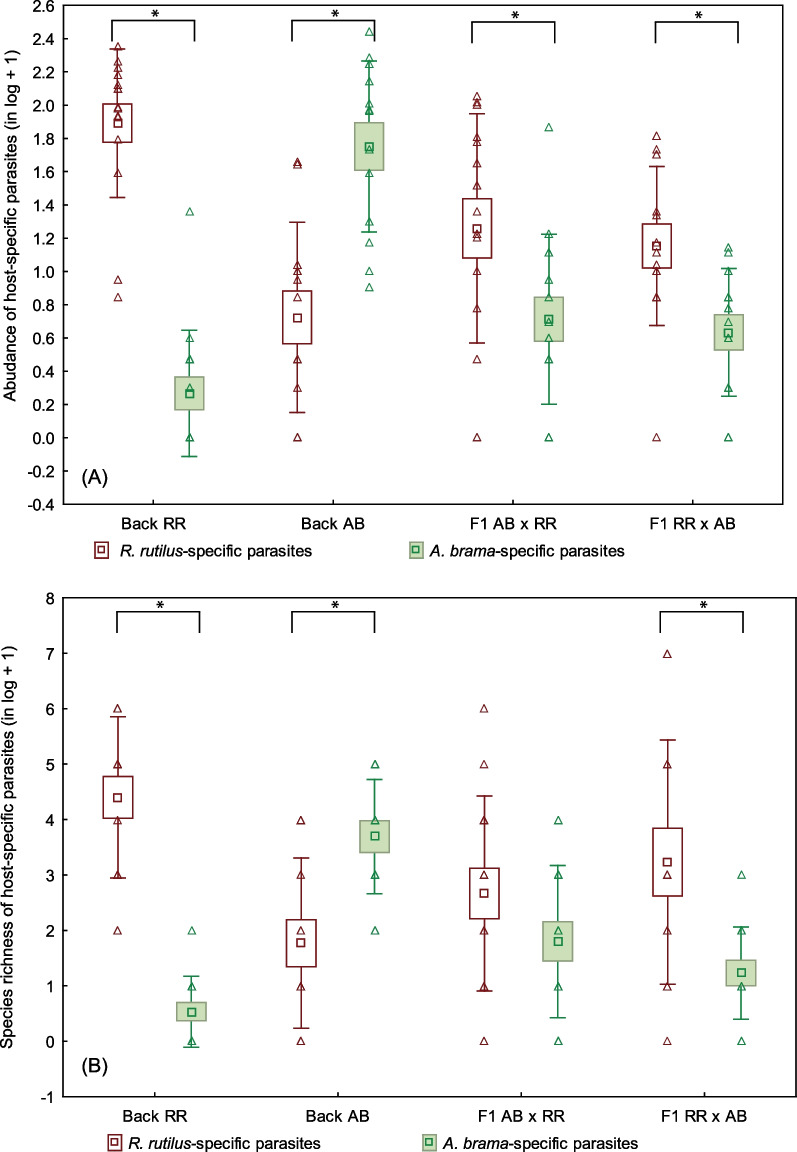
*R. rutilus*-associated and *A. brama*-associated monogenean parasites in F1 generations and backcross generations of hybrids. Monogenean abundance (**A**) and species richness (**B**) of *R. rutilus*-associated and *A. brama*-associated monogenean parasites in F1 generations of hybrids (F1 AB × RR – F1 generation with *A. brama* mtDNA, F1 RR × AB—F1 generation with *R. rutilus* mtDNA) and backcross generations of hybrids (Back RR—backcross generation with *A. brama* mtDNA and paternal *R. rutilus*, Back AB—backcross generation with *A. brama* mtDNA and paternal *A. brama*). AB—*A. brama*, RR—*R. rutilus*. Three monogenean species (*Gyrodactylus vimbi*, *G. carassi* and *Paradiplozoon homoin*) were present on both *R. rutilus* and *A. brama*, therefore these parasite species were excluded from the analyses. Significant difference between *R. rutilus*-associated and *A. brama*-associated monogenean parasites in each fish group are shown by asterisks

The abundance of roach-associated monogeneans was higher than the abundance of common bream-associated monogeneans on F1 hybrids with common bream mtDNA (Wilcoxon test, N = 14, Z = 2.919, *p* = 0.004 for abundance), but no significant difference was found between roach-associated species richness and common bream-associated species richness (*p* = 0.084) (Fig. 2). The abundance and species richness of roach-associated monogeneans were higher than those of common bream-associated monogeneans on F1 hybrids with roach mtDNA (Wilcoxon test, N = 13, Z = 2.411, *p* = 0.016 for abundance (close to the significance when applying Bonferroni correction, *p* < 0.0125) and N = 11, Z = 2.578, *p* = 0.010 for species richness) (Fig. 2). The MW test showed no effect of the mtDNA of F1 hybrids on the differences in parasite species richness or parasite abundance between roach- and common bream-associated parasites (*p* > 0.05).

The abundance and species richness of roach-associated monogeneans were higher than common bream-associated monogeneans on the backcross generation with roach in paternal position (Wilcoxon test, N = 15, Z = 3.408, *p* = 0.005 for both abundance and species richness) (Fig. 2). However, the abundance and species richness of common bream-associated monogeneans were higher than those of roach-associated monogeneans on the backcross generation with common bream in paternal position (Wilcoxon test, N = 13, Z = 2.795, *p* = 0.005 for abundance and N = 10, Z = 2.803, *p* = 0.005 for species richness) (Fig. 2). A significant effect of cyto-nuclear composition in backcross generations of hybrids on the differences in parasite species richness or parasite abundance between roach- and common bream-associated parasites was found (MW test, Z = 4.354, *p* < 0.001 for species richness and Z = 4.468, *p* < 0.001 for parasite abundance).

## Discussion

The present study focused on intergeneric hybridization between the divergent leuciscid species roach and common bream. Natural hybridization between these two species has been widely documented throughout Europe (mainly adult F1 hybrids have been documented; the occurrence of post-F1 generations is negligible), indicating the survival of the hybrids and their capacity to thrive in the same ecological conditions as parental species [27, 46, 48–51]. F1 hybrids in natural habitats express an intermediate niche breadth, and even use a broader trophic spectrum compared to their parental species [49, 51, 52]. Thus, the ecological prerequisites indicate that hybrids have the same opportunity as each of the parental species to be infected by free-living stages of various parasite species and/or that they have an even wider opportunity than each of parental species to encounter food infected by endoparasite species exhibiting a complex life cycle.

Our study aimed to investigate the extent of hybrid susceptibility to host-specific gill and fin monogeneans, this hypothesizing hybrid heterosis for F1 generations and hybrid breakdown for post-F1 generations. We analyzed two F1 generations, each with different mtDNA, and two backcross generations with the same mtDNA (this limitation was given by the presence of only F1 hybrids with *A. brama* mtDNA in nature that were used for artificial breeding in our study)—namely, the intra-mitotype backcross generation (having both parents with the same mtDNA i.e. the mtDNA of *A. brama*) and the inter-mitotype backcross generation (having parents with different mtDNA, with *R. rutilus* in the paternal position). The present study is in accordance with previous studies [26–28] supporting hybrid heterosis advantage in the case of F1 hybrids of evolutionarily closely-related or divergent cyprinoid species and also in line with the hybrid resistance hypothesis proposed by Fritz et al. [30]. Thus, the level of monogenean infection was lower in F1 hybrids when compared to each parental species, common bream and roach. Wolinska et al. [32] highlighted the dynamics of host and parasite genotypes driven by coevolutionary interactions on the basis of the Red Queen hypothesis. They suggested that parasites repetitively adapt to the most common host genotype through negative frequency-dependent selection; thus, parasite load has changed dynamically over time in the hybrids and parental (pure) species depending on their frequencies. However, Baird et al. [15] suggested that the relevance of the dynamic model scenario, proposed by Wolinska et al. [32], is probably only to the *Daphnia* system [53]. The study by Krasnovyd et al. [27] showed that hybrids of common bream and roach with low frequency (less than 1% of sampled fish) had low parasite loads when compared to frequent pure species that were also highly parasitized. Even if it is impossible to study coevolutionary dynamics of host-parasite interactions in long life-spanned organisms such are cyprinoid fish, Krasnovyd et al. [28] performed an experimental study using similar frequencies of each of the two parental species (phylogenetically closely related *A. brama* and *Blicca bjoerkna*) and their F1 hybrids to test whether or not host-specific parasites select associated host genotypes (as a consequence of evolutionary host-parasite coadaptation) under the conditions of similar probability of parasite encountering with an associated host or non-associated host (i.e. hybrid host exhibiting only the partial representation of genes from an associated host). They showed that the frequencies of hybrid and pure species genotypes had no effect on the level of infection by host-specific monogeneans, i.e. host-specific parasites prefer fully associated host genotypes (pure species) rather than hybrid host genotypes.

Theodosopoulos et al. [54] reviewed studies on the potential influences of parasites on species barriers in hybrid zones. They revealed three scenarios: (1) parasites maintain species barriers if hybrids pay higher fitness costs of parasite infection relative to parental species; (2) there is a breakdown of species barriers if hybrids pay lower fitness costs of parasite infection relative to parental species; and (3) parasites have no effect on species barriers if hybrids and parental species pay similar costs of parasite infection. Even if our results seem to support the second scenario i.e. breakdown of species barriers linked to lower fitness costs in hybrids paid to low parasite infection, the role of monogenean parasites promoting hybridization in the system of hybridizing cyprinoid fish species is unlikely because only F1 hybrids and only in very low frequencies have been mostly documented in cyprinoids [7, 27], and because monogenean parasites coevolutionary closely associated with their hosts usually do not exhibit any virulence in wild living fish hosts. In hybridizing system of common bream and roach, other multiple evolutionary and ecological mechanisms linked to breakdown of species barriers have been proposed [45–49, 55, 56].

Finally, we suggest that host-parasite coadaptation play a role in determining the low parasite infection of F1 hybrids of cyprinoid fish. Direct hybridization (resulting in the F1 generation) likely precludes high intensities of parental species-associated parasites in host genomes that are not fully co-adapted to parasite genomes [7, 27, 57]. This hypothesis may be supported also by the fact that increasing the proportion of parental taxa-associated genes in hybrid genomes (backcross generations) is associated with increasing the abundance of parental taxa-associated parasites (see below).

In the present study, almost all monogenean species associated with one or the other parental species were present on many representatives of each of the four hybrid generations investigated. Baird et al. [15] suggested a model of hybridization effects on parasite load in a hybrid zone based on the prediction that positive hybridization effects will reduce parasite load or diversity, indicating hybrid resistance, and that negative hybridization effects will increase load or diversity, indicating hybrid susceptibility. Previous studies on fish monogeneans as well as the present study consistently reveal that high parasite diversity is not a good predictor of hybrid breakdown, as F1 hybrids of cyprinoid species harbor host-specific parasite species of both parental species but with a low intensity of infection. This pattern seems to be more closely related to host-parasite genetic coadaptation (see below) than to host resistance or susceptibility. According to the presence-absence data, monogenean communities of roach and common bream were highly divergent, supporting the host specificity of their monogenean faunas. The overall similarity between different pairs of generations of hybrids, calculated using presence-absence data, was high. However, the absence of a few monogenean species was reported in hybrids. More specifically, three rare *Dactylogyrus* species associated with roach (*D. similis*, *D. rarissimus* and *D. fallax*) were absent in some hybrid generations. A parasite species associated with common bream, *D. wunderi*, was not present on hybrids of the F1 generation with roach mtDNA or on backcross hybrids with paternal roach (i.e., the backcross generation with a higher proportion of roach nDNA). The previous study by Krasnovyd et al. [27] showed that F1 hybrids of roach and common bream have limited susceptibility to common bream-specific parasites, i.e., they revealed the absence of *D. zandti* and the presence of a single specimen of *D. wunderi* on F1 hybrids under the condition of the low frequency of these hybrids in natural habitats. Limited susceptibility to common bream-specific *Dactylogyrus* was even reported in a study of phylogenetically closely-related *A. brama* and *B. bjoerkna* using an experiment with similar frequencies of each of parental species and F1 hybrids [28]. More specifically, their study showed the very limited susceptibility of F1 hybrids to common bream-associated *D. wunderi* and *D. auriculatus*, whilst this pattern was not evidenced for the two common bream-associated *Dactylogyrus* species and three silver bream-associated *Dactylogyrus* species. Our findings support the previous studies by Krasnovyd et al. [27, 28] suggesting a difference between the degrees of host-parasite coadaptation for specific monogenean parasites in the two hybridizing systems of leuciscid species, always with stronger host-parasite coadaptation in the case of common bream and its associated *Dactylogyrus* species*.*

Krasnovyd et al. [27] showed that the maternal ancestry of hybrids may influence the infection level of some parasite species; more specifically, F1 hybrids with roach mtDNA and F1 hybrids with common bream mtDNA differed in the infection of generalist digenean and crustacean species. Later, Krasnovyd et al. [28] examined the effect of the maternal ancestry of F1 hybrids (mtDNA) on the level of monogenean infection, and inferred that the maternal mtDNA of F1 hybrids of common bream and silver bream is not an important predictor of their host-specific monogenean infection, a fact which is also supported by the present study of common bream and roach hybridization.

Our study revealed similar total monogenean abundance, and a similar or slightly lower abundance of individual monogenean species in backcross generations when compared to each of the parental species, roach and common bream. This finding does not support the hybrid breakdown hypothesis, which predicts that backcross generations are more intensively parasitized than parental generations. On the basis of cyto-nuclear incompatibilities resulting from the mismatch of the mitochondrial and nuclear genomes of hybridizing species [5, 6, 21], vitality-related and fitness-related traits should be weakened in paternal (inter-mitotype) backcross generations; i.e., we expected high parasite load to be related to the cyto-nuclear incompatibility of paternal backcross hybrids. This was not the case, as a similar level of total monogenean infection was found in both intra-mitotype and inter-mitotype backcross generations, and no significant difference in total monogenean infection between backcross generations and each of the parental species was found.

Our study showed an asymmetrical distribution of parental species-associated parasite species in F1 generations of hybrids and backcross generations of hybrids. This asymmetry was reported for monogenean abundance as well as for species richness (although the difference between roach-associated species richness and common-bream associated species richness was not significant for F1 hybrids with common bream mtDNA). Both generations of F1 hybrids exhibited the same asymmetry, both being more infected by roach-associated parasites. This pattern was even previously shown by Krasnovyd et al. [27], who focused on the investigation of host-associated monogenean parasites under the conditions of a very low frequency of F1 hybrids, and a lower frequency of roach when compared to the frequency of common bream in a natural habitat. Our study clearly demonstrates that this asymmetry is not affected by the frequencies of F1 hybrid, common bream, or roach genotypes or by the intensities of monogenean species in common bream and roach (in our study, no significant difference in monogenean abundance between common bream and roach was found in contrast to the study of Krasnovyd et al. [27]).

Until now the asymmetrical distribution of parental species-associated parasites was not investigated in backcross generations of hybrids. In our study, we revealed that the backcross generation with roach in paternal position (i.e. the backcross generation exhibiting a higher proportion of nDNA from roach than from common bream and exhibiting common bream mtDNA) exhibited a pattern of asymmetry identical to that shown by F1 generations of hybrids. However, the opposite asymmetry was reported for communities of monogeneans in the backcross generation exhibiting a higher proportion of nDNA from common bream than from roach and exhibiting common bream mtDNA. This backcross generation of hybrids had a higher abundance and species richness of common bream-associated parasites when compared to roach-associated parasites.

The different asymmetrical distributions of parental species-associated parasites in two backcross generations clearly support the role of host-parasite coevolution (specifically, the role of reciprocal host-parasite coadaptation) in determining the level of host susceptibility to monogenean species associated with roach or those associated with common bream. Increasing the proportion of the nDNA of one parental taxon in the hybrid genome is clearly associated with a higher level of susceptibility to the parental taxon-associated monogenean species. Nevertheless, we should admit that there is some limitation of our study as only two backcross generations (both with common bream mtDNA) were analyzed which was related to the fact that only F1 hybrids with common bream mtDNA useful for our artificial hybridization experiment were found in natural habitats. To clarify whether or not cyto-nuclear incompatibilities in hybrid genomes may play some role in enhancing infection by parental species-specific parasites, future studies comparing all four potential backcross generations representing different combinations of the nDNA and mtDNA of both parents will be necessary. However, we proposed that the level of infection by host-specific parasites in intergeneric hybrids of fish is strongly limited by fish genetic background, i.e., the extent of parental species genes present in the hybrid genome, as the genome of pure (parental) species is coevolutionarily associated with specific parasite species. If our hypothesis is correct, we can even doubt whether or not hybrid heterosis is the main mechanism driving infection by host-specific parasites in F1 generations of hybrids.

## Conclusions

We can summarize that F1 and post-F1 generations of intergeneric fish hybrids harbored almost all monogenean species specific to one or specific to the other parental taxon. F1 and post-F1generations differed in their levels of monogenean infection. Whist the parasite infection in F1 generations was in line with the hybrid heterosis hypothesis and the hybrid resistance scenario, post-F1 generations, i.e. backcross generations, were more heavily infected than F1 generations. The presence of different asymmetrical distributions of parental taxa-associated parasites in the two backcross generations is more consistent with host-parasite coadaptation than with hybrid breakdown. Comparisons of intra-mitotype and inter-mitotype backcross generations did not reveal the role for genetic incompatibilities in determining hybrid vigour, estimated using the level of monogenean infection. We strongly suggest the need for future studies focusing on potential genes involved in fish host-monogenean coadaptation.

## Material and methods

### Experimental fish lines

Specimens of *A. brama, R. rutilus*, and their F1 hybrids were collected from the Hamry Reservoir (49.73724N, 15.91395E; the Czech Republic) and transported to the breeding facility. All F1 hybrids exhibited *A. brama* mtDNA. The hybrids were identified using meristic traits (i.e. the number of gill rakers, the number of scales in the lateral line, and the number of branched rays in the anal fin) and molecular markers (the cytochrome *b* gene and microsatellite loci following Krasnovyd et al. [27]).

The fish were separated according to sex into two well-aerated tanks and stimulated for ovulation/spermiation by carp pituitary (females received 2 doses, 0.3 and 2.7 mg/kg, 24 h and 12 h before propagation, respectively; males received 1 mg/kg 24 h before propagation) and by subsequently increasing the water temperature to 22 °C. Oocytes of ovulating females were obtained by the dry method and sperm was sampled according to Linhart et al. [58]. Hatchery water was used for gamete activation.

Artificial spawning based on individual pair mating was performed using the following parental combinations: (1) *A. brama* female and male (2) *R. rutilus* female and male, (3) *A. brama* female and *R. rutilus* male, (4) *R. rutilus* female and *A. brama* male, (5) hybrid (F1) female and *A. brama* male, and (6) hybrid (F1) female and *R. rutilus* male. As only natural F1 hybrids with *A. brama* mtDNA were identified in natural habitat and were used for artificial crosses, this study is limited only to the two backcross generations, i.e. backcross generation resulting from the crosses of both parents with *A. brama* mtDNA (female F1 hybrid with *A. brama* mtDNA and *A. brama* male), and backcross generation resulting from the crosses of parents with different mtDNA (female F1 hybrid with *A. brama* mtDNA and *R. rutilus* male).

Fish representing 6 fish lines were reared to the age of 2 years. The following fish lines were used in the analyses (sample size and total body weight in g (mean ± SD) are included with each line): pure *R. rutilus* 9.69 ± 2.59 (18 specimens), pure *A. brama* 9.70 ± 4.88 (13 specimens), F1 *A. brama* x *R. rutilus* 9.68 ± 4.22 (15 specimens), F1 *R. rutilus* × *A. brama* 8.15 ± 4.04 (13 specimens), backcross of F1 hybrid x *R. rutilus* (termed backcross with roach in paternal position) 9.01 ± 3.27 (15 specimens), and backcross of F1 hybrid × *A. brama* (termed backcross with common bream in paternal position) 7.91 ± 4.70 (13 specimens).

### Monogenean infection and parasite collection

We performed co-habitation experiment following Krasnovyd et al. [28] i.e. experimental parasite-free fish specimens resulting from artificial breeding were placed into the same tank together with infected donor specimens (*R. rutilus* and *A. brama*). Specifically, fish were placed in one experimental tank containing specimens of all experimental six lines and the infected parental taxa (*R. rutilus* and *A. brama*) i.e. specimens of parental taxa collected from the locality with their natural distribution. Specimens of experimental lines of hybrid generations were labelled individually by cutting a small part of different fins as follows: a small part of caudal fin for F1 *A. brama* × *R. rutilus*, a small part of dorsal fin for F1 *R. rutilus* × *A. brama*, a small part of left pectoral fin for backcrosses of F1 hybrid × *R. rutilus,* and a small part of right ventral fin for backcrosses of F1 hybrid × *A. brama*. Prior to experimental infection, some wild living specimens of *R. rutilus* and *A. brama*, i.e. specimens cached in their natural habitat, were examined for the presence of monogenean species (gill and fin parasites with a direct life cycle). These fish specimens served as the source of infection by monogenean parasites exhibiting direct life cycle strictly associated with water environment (oviparous *Dactylogyrus* spp. and *Paradiplozoon* spp. and viviparous *Gyrodactylus* spp.) for the 6 experimental lines of fish.

Fish specimens i.e. specimens of six lines obtained by artificial breeding were dissected using the standard method described by Řehulková et al. [59] and all monogenean species including *Dactylogyrus, Gyrodactylus*, and *Paradiplozoon* were collected. More precisely, monogenean specimens were removed from the gills and fins, mounted on slides, and fixed using a mixture of glycerin and ammonium picrate (GAP) for further determination. Species identification was performed using an Olympus BX51 microscope equipped with phase contrast optics. All *Dactylogyrus* spp.*, Gyrodactylus* spp., and *Paradiplozoon* spp. were identified using Moravec [43]; more specifically, the determination was based on the sclerotized parts of the parasite haptor and, for *Dactylogyrus* species, also on the sclerotized parts of the reproductive organs.

Prevalence (the percentage of infected fish in each sample), abundance (the number of parasite specimens per host taking into account both infected and uninfected hosts) and intensity of infection (the number of parasite specimens per infected host) were calculated for each parasite species in each host group following Bush et al. [60].

### Statistical analyses

The Mann–Whitney (MW) test was used to test differences in the abundance and intensity of infection of each parasite species between each of the parental species (roach or common bream) and each of the hybrid groups (the F1 generation with roach mtDNA, the F1 generation with common bream mtDNA, the backcross generation with common bream mtDNA and roach in paternal position, and the backcross generation with common bream mtDNA and common bream in paternal position). Differences in prevalence between each parental species and each hybrid group were tested using the Fisher exact test. Bonferroni correction was applied for multiple tests.

The Kruskal–Wallis (KW) test with multiple comparisons was used to test the differences in total monogenean abundance or species richness among roach, common bream, and four groups of hybrids. Bonferroni correction was applied for multiple tests.

The Wilcoxon pair test was used to analyze the asymmetrical distribution of roach-associated and common bream-associated monogenean species in F1 generations of hybrids and backcross generations of hybrids. We defined a roach-associated parasite as a parasite species present on roach but absent on common bream, and a common bream-associated parasite as a parasite present on common bream but absent on roach. The MW test was applied to test the effect of mtDNA on parasite abundance or species richness in F1 hybrids. The MW test was applied to test the effect of different cyto-nuclear combinations (i.e. parents sharing the same mtDNA versus parents exhibiting different mtDNA) on parasite abundance or species richness in backcross hybrids. To test the effect of the mtDNA of F1 hybrids on the asymmetrical infection of parental species-associated monogeneans, the difference between roach-associated parasite species richness (or abundance) and common bream-associated parasite species richness (or abundance) was calculated and used as a dependent variable in the MW test. The same approach was applied to test the effect of different cyto-nuclear combinations on the asymmetrical infection of parental species-associated monogeneans in backcross hybrids. Bonferroni correction was applied for multiple tests.

Statistical analyses were performed in Statistica 14.0 for Windows (TIBCO software Inc., Palo Alto, CA, USA).

The Jaccard index was used to calculate similarity in parasite communities between pairs of generations based on the presence-absence data. The calculation was performed in Past 2.16 [61].

## Data Availability

Not applicable.
